# Effects of Extraction Buffer on the Solubility and Immunoreactivity of the Pacific Oyster Allergens

**DOI:** 10.3390/foods10020409

**Published:** 2021-02-12

**Authors:** Roni Nugraha, Thimo Ruethers, Elecia B. Johnston, Jennifer M. Rolland, Robyn E. O’Hehir, Sandip D. Kamath, Andreas L. Lopata

**Affiliations:** 1 Department of Aquatic Product Technology, Faculty of Fisheries and Marine Science, IPB University, Bogor 16680, West Java, Indonesia; rnugraha@apps.ipb.ac.id; 2 Molecular Allergy Research Laboratory, College of Public Health, Medical and Veterinary Sciences, James Cook University, Douglas, QLD 4811, Australia; thimo.ruethers@my.jcu.edu.au (T.R.); elecia.johnston@jcu.edu.au (E.B.J.); sandip.kamath@jcu.edu.au (S.D.K.); 3 Australian Institute of Tropical Health and Medicine, James Cook University, Douglas, QLD 4811, Australia; 4 Centre for Sustainable Tropical Fisheries and Aquaculture, Faculty of Science and Engineering, James Cook University, Douglas, QLD 4811, Australia; 5 Centre for Food and Allergy Research, Murdoch Children’s Research Institute, Parkville, VIC 3052, Australia; 6 The Department of Immunology and Pathology, Monash University, Clayton, VIC 3800, Australia; jennifer.rolland@monash.edu (J.M.R.); robyn.ohehir@monash.edu (R.E.O.); 7 The Department of Allergy, Immunology and Respiratory Medicine, The Alfred Hospital and Monash University, Melbourne, VIC 3004, Australia

**Keywords:** allergens, extraction buffer, proteomics, allergenomics, shellfish, immunoreactivity, mollusk allergy, Pacific oyster

## Abstract

Despite recent technological advances, novel allergenic protein discovery is limited by their low abundance, often due to specific physical characteristics restricting their recovery during the extraction process from various allergen sources. In this study, eight different extraction buffers were compared for their ability to recover proteins from Pacific oyster (*Crassostrea gigas*). The protein composition was investigated using high resolution mass spectrometry. The antibody IgE-reactivity of each extract was determined using a pool of serum from five shellfish-allergic patients. Most of the investigated buffers showed good capacity to extract proteins from the Pacific oyster. In general, a higher concentration of proteins was recovered using high salt buffers or high pH buffers, subsequently revealing more IgE-reactive bands on immunoblotting. In contrast, low pH buffers resulted in a poor protein recovery and reduced IgE-reactivity. Discovery of additional IgE-reactive proteins in high salt buffers or high pH buffers was associated with an increase in allergen abundance in the extracts. In conclusion, increasing the ionic strength and pH of the buffer improves the solubility of allergenic proteins during the extraction process for oyster tissue. This strategy could also be applied for other difficult-to-extract allergen sources, thereby yielding an improved allergen panel for increased diagnostic efficiency.

## 1. Introduction

Shellfish allergy is an emerging chronic disease affecting up to 10.3% of the general population [1,2]. Shellfish allergy is caused by overreaction of the human immune system to harmless shellfish proteins resulting in allergic sensitization and a range of different clinical presentations including urticaria (hives), angioedema (swelling of throat or other tissues), bronchospasm (trouble breathing), hypotension (low blood pressure and dizziness), and even life-threatening anaphylaxis or occasionally death [2]. Upon subsequent exposure to the human immune system, allergenic proteins trigger the production of more allergen-specific IgE antibodies, which bind to specific receptors on the surface of mast cells and basophils. When these allergenic proteins bind to receptor-bound antibodies, subsequent cross-linking results in activation of these cells leading to mediator release and clinical symptoms [3]. Currently, over 2000 allergenic proteins have been identified, and almost 1000 are analysed in detail and have been assigned a unique code by the WHO (World Heatlh Organization)/IUIS (International Union of Immunological Societies) Allergen Nomenclature Sub-committee [4].

Previously, bioinformatics analysis of the Pacific oyster genome identified the transcripts of 95 potential allergens [5]. These proteins belong to known protein families including various allergenic proteins, and the amino acid sequence similarity with their homologous allergens is very high. However, after proteomic analysis of protein extracts from the oyster, it was observed that not all identified potential allergens were present in the extract using traditional phosphate buffer. The shortcoming of extractability of commonly used buffers, such as phosphate-buffered saline (PBS) or tris-buffered saline (TBS) has been shown in several studies. Cardona et al. [6] could not obtain any allergens from mango extracted using TBS without additional treatments. Similarly, paramyosin was overlooked during IgE-binding analysis of abalone *Haliotis discus discus* proteins extracted using PBS but was observed after increasing the sodium chloride (NaCl) concentration in the buffer to up to 0.9 M [7]. This highlights the importance of an optimal extraction method for a specific allergen source and implementation of the appropriate buffering system for maximum recovery of allergens.

Several studies compared different factors that are known to influence extractability of proteins to optimize the extraction of allergens from different food sources such as peanut [8,9] and shrimp [10]. Most studies, however, focused on the extraction of the major allergens, and thus omitted to study the presence of other allergens that also contribute to the allergic reaction. Unlike shrimp or other shellfish species, the bivalve mollusk oyster is often consumed raw. It is, therefore, of particular importance to investigate the effect of buffer composition on the protein and allergen content of both raw extracts and heated extracts.

## 2. Materials and Methods

### 2.1. Preparation of Extraction Buffers

To determine the effects of extraction buffers on the composition of soluble proteins, eight different buffers were prepared for comparison (Table 1). Phosphate-buffered saline (PBS) and Tris-buffered saline (TBS) buffers with low ionic strength, pH 7.4, were included as internal controls since they are the most frequently used buffers for the extraction of proteins. Sodium chloride was used as an additive for the PBS and TBS buffers to prepare high ionic strength buffers. The low ionic strength TBS and PBS buffers contained 137 mM NaCl, while the high ionic strength buffers contained 1 M NaCl. Carbonate buffers with generally high pH are commonly used as coating or coupling buffers in enzyme-linked immunosorbent assay (ELISA) and lateral flow device (LFD) development. Therefore, they were included in this investigation to determine the effect of higher pH. Citrate buffers were chosen as low-pH buffers to cover a wider pH range for the investigation.

### 2.2. Preparation of Oyster Soluble Protein Extracts

Five grams of minced fresh Pacific oysters (*Crassostrea gigas*) purchased from a local market in Townsville, Australia were added to 25 mL of each extraction buffer and homogenised using a T 10 basic ULTRA-TURRAX disperser (IKA, Humboldtstraße 8, 53, 639 Königswinter, Germany) and subsequently stirred overnight at 4 °C. The extracts were centrifuged at 15,000 × *g* for 15 min, and the clear supernatant was further filtered through a 0.45 µm membrane to attain the final extracts. These extracts were designated as raw extracts. Meanwhile, heated extracts were obtained by heating an aliquot of the raw extracts at 100 °C for 15 min in a water bath. It was ensured that the tissue extract slurry achieved a final temperature of 100 °C. These extracts were then centrifuged and processed as above [5]. All extracts were stored at −20 °C until further analysis.

### 2.3. Quantification of Protein Content

The concentration of protein in each extract was estimated using the bicinchoninic acid assay (BCA) kit (Pierce Biotechnology Inc., Rockford, IL, USA) following the protocol as described previously [11].

### 2.4. Proteomic Profiling of Oyster Extracts

The protein composition of each extract was identified using the shotgun mass spectrometry analysis. Gel-aided sample preparation (GASP) technique was used to prepare the samples following the procedure described by Fischer and Kessler [12]. Fifty microlitres of solution of 100 µg of proteins was denatured for 20 min in the presence of 50 mM of dithiothreitol (DTT) to reduce disulfide bridges. An equal volume of 40% acrylamide-bis solution (37.5:1) (Merck, VIC, Australia) was added, mixed gently and left at room temperature for 20 min. Subsequently, 5 µL of tetramethylethylenediamine (TEMED) and 5 µL of 10% ammonium persulfate (APS) were added and left at room temperature to initiate polymerisation. The gel plug was removed upon the completion of polymerisation, and transferred to a minicolumn (Promega, Alexandria, NSW, Australia) in which the filter membrane had been removed previously by dissolving in acetone. A solution containing methanol/acetic acid/water (50/40/10) was added to fix the gel pieces. The proteins were then digested following the protocol described [5]. After proteolytic digestion, the peptide solutions were desalted using C18 ZipTip^®^ pipette tips (Millipore, Billerica, MA, USA) dried under vacuum, resuspended in 20 μL 0.1% formic acid and then subjected to liquid chromatography tandem mass spectrometry (LC MS/MS) analysis.

### 2.5. Mass-Spectrometry Analysis and Protein Identification

The eluted peptides were analysed with an LTQ Orbitrap Elite (Thermo Fisher Scientific, Melbourne, VIC, Australia) with a Nano ESI interface in conjunction with an Ultimate 3000 RSLC nano-HPLC (Dionex Ultimate 3000, Thermo Fisher Scientific, Melbourne, VIC, Australia) at the Bio21 Institute, Melbourne, Australia following the procedure described in [5]. Label free quantification was conducted for the proteins in each extraction buffer using MaxQuant 1.6.5.0 [13] complemented with the Andromeda Search engine and searched against the in-house database of the oyster proteome downloaded from the UniProt (https://www.uniprot.org/proteomes/UP000005408). The approximate abundance of the proteins was calculated using iBAQ algorithm [14], which measures the intensity of each protein by summing up the precursor peptides of that protein and dividing it by the number of theoretically observable peptides. The absolute amount of each protein in each extract was determined by dividing the protein’s iBAQ value by the sum of all non-contaminant iBAQ values, generating an riBAQ value for each protein and a normalized measure of molar abundance (relative iBAQ):

(1) $$\mathrm{riBAQ}\;=\;\frac{{\mathrm{iBAQ}}_{i}}{\sum_{i\;=\;1}^{n}{\mathrm{iBAQ}}_{i}}$$

### 2.6. SDS–PAGE and IgE-Reactive Analysis of Oyster Extracts

The protein components of extracts were profiled using sodium dodecyl sulfate-polyacrylamide gel electrophoresis (SDS-PAGE) according to the method of Laemmli [15]. A solution of each extract containing 10 µg of protein was mixed with Laemmli buffer and heated at 95 °C for 5 min. The solution was loaded onto each of the wells of SDS-acrylamide gel and the proteins were separated at 170 V for 1 h. The resolved protein bands on the gel were stained with Coomassie Brilliant Blue and visualised using the Odyssey^®^ CLx Imaging System (LI-COR Biosciences, Lincoln, NE, USA) [16].

For IgE binding analysis, after the electrophoresis was completed, the separated proteins were transferred to a nitrocellulose membrane using a Trans-Blot^®^ SD Semi-Dry Electrophoretic Transfer Cell (BioRad, Hercules, CA, USA). Subsequently, the membrane was blocked using Casein blocking solution (Sigma, St. Louise, MO, USA) for 1 h at room temperature. The blocked-nitrocellulose membrane was incubated overnight with a pooled serum from five shellfish-allergic patients (Table 2) diluted 1:20 in PBST with added casein. After the washing step, secondary anti-human IgE (1:10,000 dilution, DAKO Corporation, Lincoln, NE, USA) was added and incubated for 1 h. The membrane was subsequently incubated for 35 min with donkey anti-rabbit IgG antibody conjugated infrared IRDye 800CW (1:10,000 dilution, LI-COR, Lincoln, NE, USA), and IgE antibody binding was visualised using the Odyssey^®^ CLx Imaging System (LI-COR Biosciences, Mulgrave, VIC, Australia) [17]. IgE reactive spots were annotated to the protein profile on SDS-PAGE, and corresponding bands cut out, tryptic digested and analysed using mass spectrometry. Identification of the proteins was carried out using the Mascot search engine and cross-referenced against the in-house database of the oyster proteome downloaded from UniProt (https://www.uniprot.org/proteomes/UP000005408), supplemented with sequences from the common Repository of Adventitious Proteins (https://www.thegpm.org/crap/) [5]. 

### 2.7. Statistical Analysis and Experimental Design

The extraction processes were conducted in triplicate. Differences in protein content of each extract were examined by analysis of variance (ANOVA) using Prism (version 7.03, 2017, GraphPad Software Inc., La Jolla, CA, USA). The Tukey test was used for comparison of the means. The level of significance was set at *p* < 0.05.

## 3. Results

### 3.1. Effects of Extraction Buffers on Soluble Protein Content

The quantification of protein content for each extract clearly showed that the number of soluble proteins varied greatly (*p* < 0.05, Table 3). High pH buffers were able to extract a significantly higher concentration of proteins than low pH buffers. The carbonate-10 buffer demonstrated the best extraction properties resulting in 10.4 mg/mL of extracted proteins. The carbonate-9 buffer, however, did not differ greatly to the control PBS in its ability to extract proteins (8.0 and 7.7 mg/mL proteins, respectively, *p* > 0.05), while the control TBS resulted in slightly lower protein yield although not significantly different (7.0 mg/mL of protein, *p* > 0.05). Both citrate buffers at low pH showed poor extraction properties, resulting in only 2.3 mg/mL and 3.0 mg/mL of proteins, respectively. Addition of salt up to 1 M to the PBS and TBS buffers significantly increased the ability of the buffers to retrieve soluble proteins (*p* < 0.05).

The outcomes of using different buffers were clearly distinct when extracted proteins underwent heat-treatment in each of the corresponding buffers. The distribution of protein concentrations of the heated extracts was different to the raw extracts. Instead of higher-pH buffers resulting in higher concentration of protein and vice versa, the concentrations of proteins were consistently low across all buffers. While most of the proteins heat-treated in TBS, PBS and carbonate buffers were either degraded or aggregated resulting in decreased protein content, the protein concentration for the citrate buffers remained the same, possibly indicating that there was no protein loss. Heat-treatment reduced up to 80% of the protein content in the TBS, PBS and carbonate-9 buffers and up to 60% in the carbonate-10 buffer. A higher ionic strength in buffers did result in an increased number of recovered proteins as seen in both PBSN and TBSN when compared to PBS and TBS, however this was statistically not significantly different (*p* > 0.05).

### 3.2. Proteomic Analysis of the Extracts

**Figure 1:**
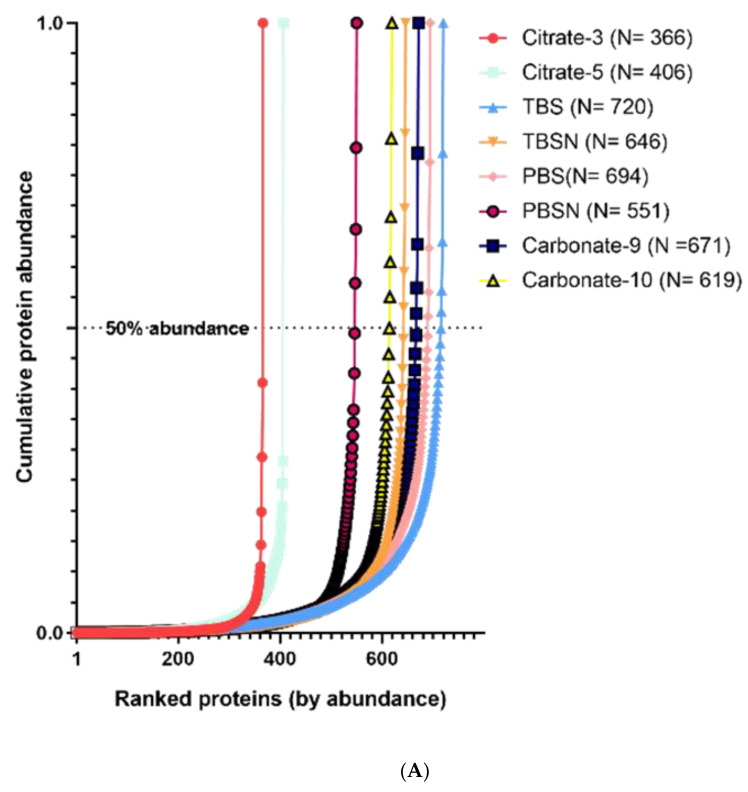

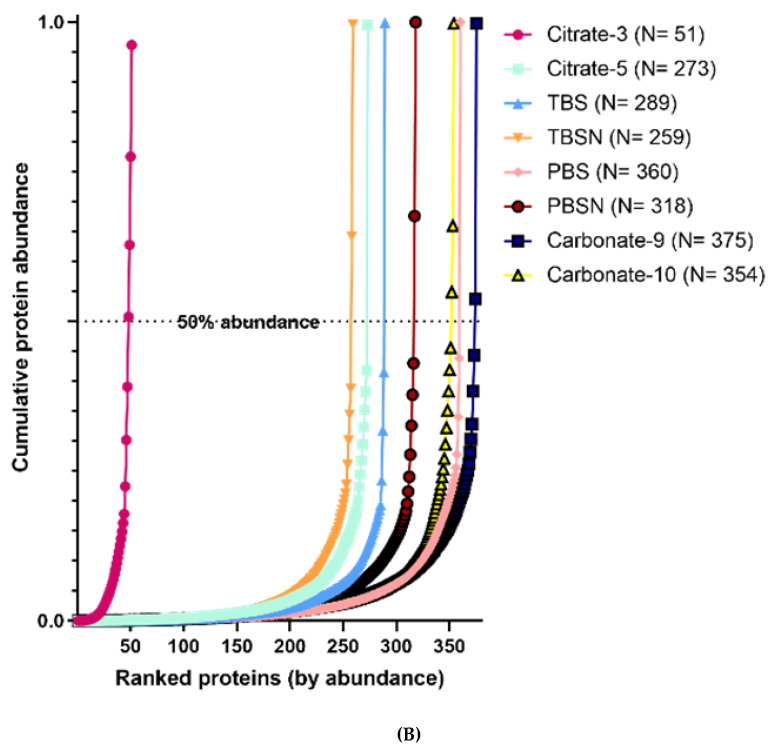
The cumulative protein mass from lowest to highest abundant proteins in relation to their mass contribution to the extract proteome in raw (**A**) and heated extract (**B**) in each buffer. Numbers in the brackets indicate the number of proteins identified.

To identify the proteins recovered by each buffer, a shotgun proteomic approach using gel-aided sample preparation (GASP) was applied. GASP is a simple, robust and well-established protocol for in-gel sample preparation without the need of alkylation, precipitation, filtering or electrophoresis steps [12,18]. The peptide spectra were processed using the MaxQuant platform with an MS label-free method adapted for protein quantification. The protein abundance was calculated by applying the iBAQ methodology, which has been described to have a good correlation with known relative protein amounts over at least four orders of magnitude [14]. This method estimates protein abundance as the sum of intensities of all tryptic peptides identified for each protein divided by the theoretically observable peptides, obtained by in silico digestion, taking into account only peptides consisting of 6–30 amino acid residues. In the current study, the resulting iBAQ intensities were used to provide an accurate determination of the relative abundance of all identified proteins. The number of proteins identified in each raw extract differed significantly, with the lowest numbers in Citrate buffers. As low as 366 and 406 proteins were identified in Citrate-3 and Citrate-5 buffer, respectively (Figure 1). Interestingly, the number of proteins in TBS and PBS buffer were higher than for other buffers, with as high as 720 and 694 proteins recovered, respectively. Cumulative riBAQ values show that few proteins contributed to more than 50% of proteome abundance. 60S ribosomal protein L40, structural constituent of ribosome, demonstrated an riBAQ value of over 0.5 in Citrate buffers and was found as the most abundant protein as well as in TBS, PBS, PBSN and Carbonate-9 buffers. Meanwhile, actin was found as the most abundant protein in TBSN and Carbonate-10 buffers. Heat treatment had significant effects on the proteome of Pacific oyster in all extraction buffers, particularly the Citrate-5, which experienced significant loss of proteins. More than 50% of proteins could not be recovered for most buffers and consequently, the riBAQ values of some proteins increased greatly. In contrast to the raw extracts, only one protein, cavortin, contributed to the 50% abundance of proteome in heated preparations extracted using Citrate-5, TBS and PBS.

**Figure 2:**
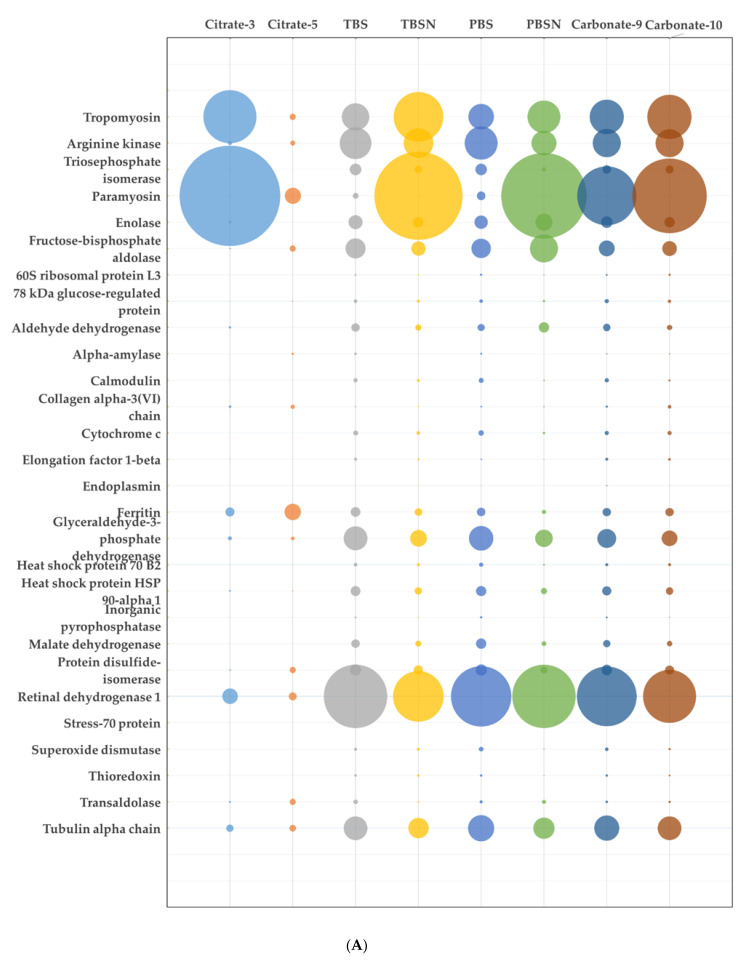

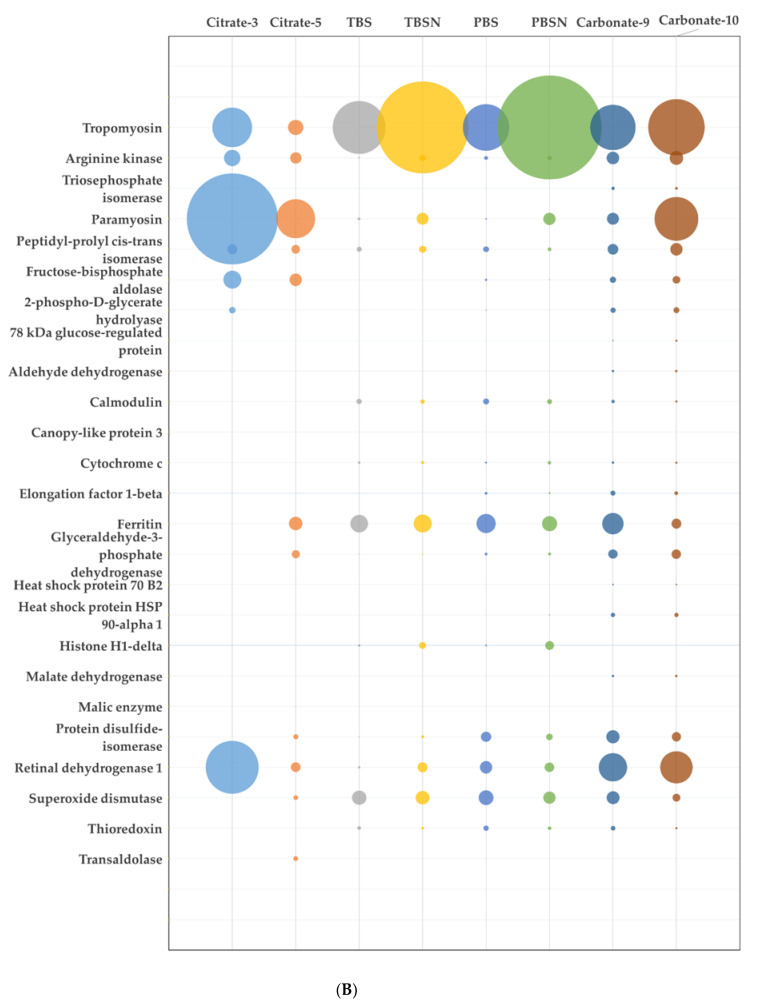
Relative abundance of potential allergens in each extraction buffer for raw (**A**) and heated extract (**B**). Sizes of the bubbles indicate the abundance of the potential allergens.

Previous research identified 95 potential allergens in Pacific oyster using bioinformatics analysis, however only some of these potential allergens were detected using proteomics analysis [5]. In order to highlight the differences in the relative abundance of each identified potential allergen from the Pacific oyster in each extraction buffer, the riBAQ values were plotted as reported in Figure 2. Variability in the abundance was observed for several potential allergens. Low pH buffers had a detrimental effect on protein solubility, particularly allergens from the cytoplasmic group such as enzymes. Myofibril proteins were very well extracted with buffers of high ionic strength and high salt buffers. Paramyosin content in raw extract increased drastically when high salt as well as high pH buffers were used for extraction, while tropomyosin slightly increased. Retinal dehydrogenase 1 was very abundant in TBS and PBS buffers, and slightly decreased in TBSN buffer as compared to TBS and PBS. The composition of recovered potential allergens in the heated extracts was very different compared to the raw extracts. Paramyosin, which was very abundant in most raw extracts, showed loss in solubility and content in the heated extracts. Very low recovery particularly in the TBS and PBS extracts was shown. Tropomyosin, as expected by its helical structure, could withstand the heat treatment and the addition of salt improved solubility.

### 3.3. Protein Profiling by SDS-PAGE

**Figure 3:**
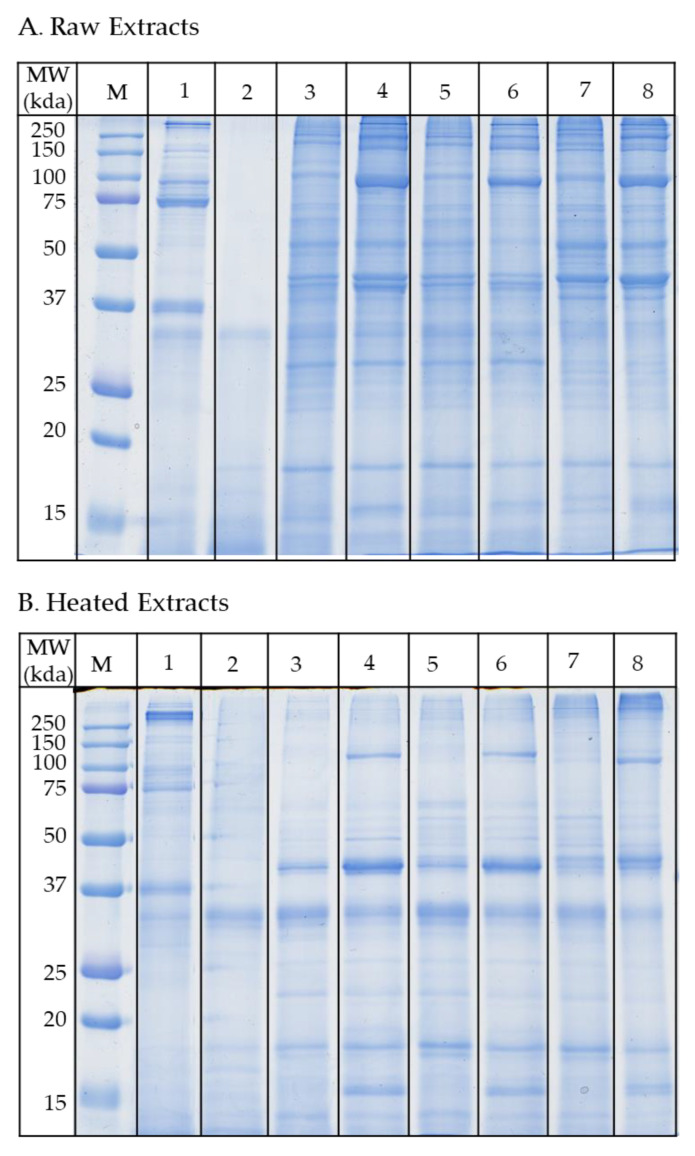
SDS-PAGE analysis of proteins from (**A**) raw and (**B**) heated oyster extracts. Samples containing 10 µg of proteins were resolved in 12% SDS-acrylamide gels and run at 170 V for 1 h. M = Marker, 1 = Citrate-3 extract, 2 = Citrate-5 extract, 3 = TBS extract, 4 = TBSN extract, 5 = PBS extract, 6 = PBSN extract, 7 = Carbonate-9 extract and 8 = Carbonate-10 extract.

The protein composition of each extract was profiled using 12% SDS-acrylamide gels under denaturing conditions (Figure 3). The raw protein profiles did not vary much between PBS, TBS and carbonate buffers; however, different intensities were observed for some bands particularly at 40 and 100 kDa (Figure 3A). The citrate buffers showed very distinct protein profiles particularly the Citrate-5. Extracts from the Citrate-3 buffer showed strong protein bands between 70–80 kDa, as well as a prominent 36 kDa band. Although the Citrate-5 contained a similar amount of protein with the citrate-3 buffer, the protein profile was very different. All bands seen in Citrate-5 buffer appeared diffused; particularly proteins above 35 kDa were absent. The protein profiles in the heated extracts were less complex than those of the raw extracts (Figure 3B). While most of the high molecular weight proteins are absent after heat treatment in most buffers, some lower molecular weight proteins (15 and 18 kDa) emerged with more intense bands. The proteins at 39 kDa, corresponding to the molecular weight range of tropomyosin, also showed more intense bands.

### 3.4. Effect of Extraction Buffers on the Presence of IgE-Binding Proteins

**Figure 4:**
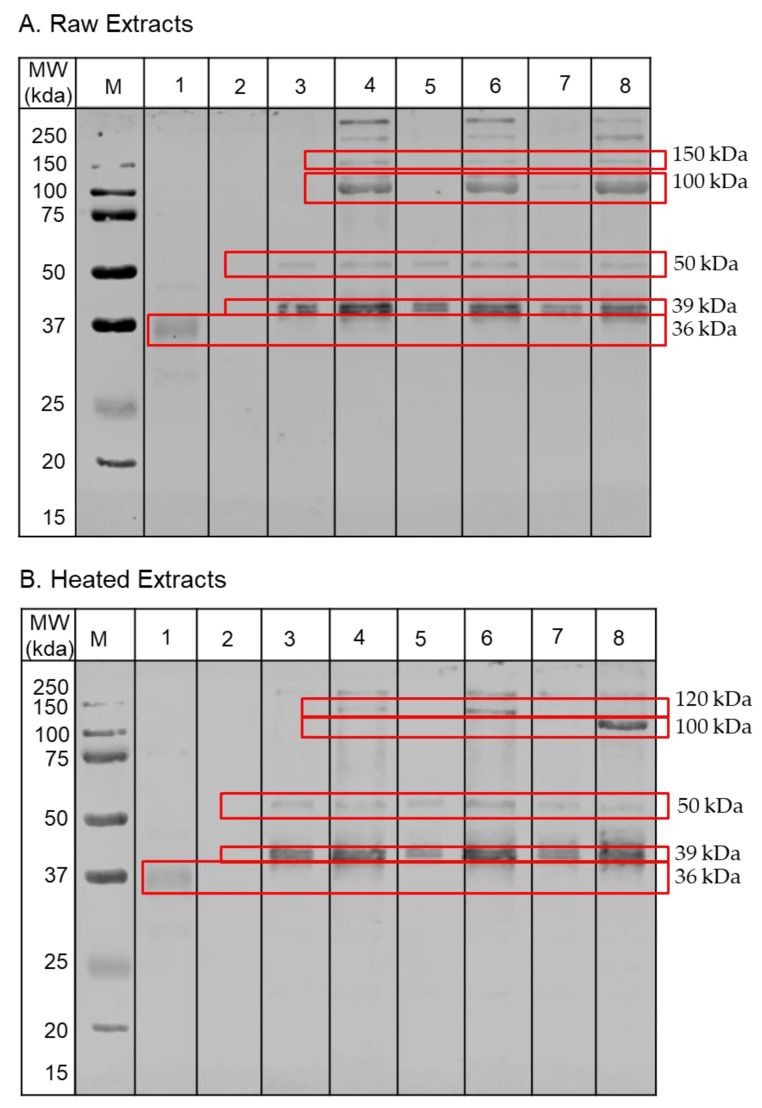
IgE-immunoblotting analysis of the proteins from (**A**) raw and (**B**) heated extracts using a pool of serum from five shellfish-allergic patients. M = Marker, 1 = Citrate-3 extract, 2 = Citrate-5 extract, 3 = TBS extract, 4 = TBSN extract, 5 = PBS extract, 6 = PBSN extract, 7 = Carbonate-9 extract and 8 = Carbonate-10 extract.

To determine whether the buffers affect the presence of the allergens in the protein extracts, immunoblotting against a pool of serum from five shellfish-allergic patients was conducted. Figure 4 shows the different profiles of IgE-reactive bands observed for both raw and heated extracts. For the raw extracts, both PBS and TBS extracts showed three prominent bands (at 36, 39 and 50 kDa). Additional strong IgE-reactive bands at high molecular weight regions (100, 120, 150 and 250 kDa) were observed with the PBSN and TBSN extracts as well as Carbonate-10 buffer extract. The Citrate-3 buffer extract showed weak IgE-bands at 36 kDa and 48 kDa while no IgE-reactivity was detected for the extract of Citrate-5 buffer.

Similarly, different patterns of IgE reactivity were observed between the raw and heated extracts. PBSN, TBSN and Carbonate-10 buffers achieved more IgE-reactive bands compared to the other buffers. Extracts from those buffers showed five IgE-reactive bands at 39 kDa, 40 kDa, 50 kDa, 120 kDa and >200 kDa. Meanwhile, the TBS, PBS and Carbonate-8 extracts lacked the IgE reactive bands at the high molecular weight position. Citrate buffers clearly had a negative impact on the extractability of allergenic proteins from the Pacific oyster as only one IgE-reactive band was observed in the Citrate-3 extract and none in the Citrate-5 extract.

To identify the proteins responsible for the IgE reactivity, selected SDS-PAGE bands at each molecular weight were cut out and tryptic digested. The top three protein families from Mascot search engine results are listed in (Table 4). In total, 11 distinct proteins were identified in the raw extracts and 6 proteins were in the heated extracts. Tropomyosin was identified at 39 kDa in both the raw and heated extracts, except in the Citrate-3 extract where the protein was identified at 36 kDa. In addition, previously identified Pacific oyster allergens [5] including arginine kinase (40 kDa), retinal dehydrogenase I (50 kDa), aldehyde dehydrogenase (50 kDa) and paramyosin (75 and 100 kDa) were detected in the raw extracts. Interestingly, paramyosin was also observed in the Carbonate-10 heated extracts. Furthermore, myosin heavy chain, a previously identified allergen in other molluscs [19,20], as well as filamin and troponin C, identified allergens in crustacean [21,22], were also detected. The other proteins including tubulin α-1C chain, α-actinin, spectrin-α chain, clathrin heavy chain, non-neuronal cytoplasmic intermediate filament protein and adipophilin were identified in the Pacific oyster IgE-reactive spots for the first time.

## 4. Discussion

Tris-based (TBS) and phosphate-based (PBS) buffer systems prepared at neutral pH (7.4) are most commonly used for the extraction of allergenic proteins from various sources. However, it was demonstrated previously that not all allergens present in the genome and transcriptome of oyster are detected in the extracted proteome [5]. These problems are also reported in previous studies on peanuts, tree nuts and venoms, showing that some allergens could not be recovered using those common buffers [5,23,24,25]. Thus, allergens are often overlooked during the discovery of novel and/or undiscovered allergens. In the current study, eight different buffers were evaluated for their capacity to extract 95 previously identified potential allergens from the Pacific oyster (*Crassostrea gigas*). The buffers were prepared to cover a wide pH range of pH 3 to 10. The effect of high concentrations of salt to the tris-based and phosphate-based buffers was also assessed. The protein recovery was compared as well as the soluble protein profile by SDS-PAGE, IgE-reactivity with patient serum as well as the protein compositions determined using mass spectrometric analysis.

The analysis of the raw extracts demonstrated a significant increase in the content of total soluble proteins using high pH buffers for the extraction as compared to the general buffers, TBS and PBS. In contrast, low pH buffers resulted in poor protein extractability, with the protein contents 3-fold lower compared to that of TBS or PBS buffer. A similar impact of the pH on the variability of recovered proteins was also observed during extraction of raw samples from peanut [8,9] and tree nuts [26]. Addition of salt to the TBS and PBS buffer improved the solubility of proteins and therefore it significantly increased the protein content in the extract. It is known that protein solubility is affected by a complex interplay between the properties of proteins, electrostatic charges and the pH of the buffers. High pH buffers change the charge of proteins to be more negative, thereby increasing water binding capacity and improving solubility of the proteins [27]. Salts are thought to play a role in improving the extractability of the buffers by associating with the opposite charges on the protein surfaces [28].

Heat treatment of the raw extracts resulted in different effects on each extract. While a significant reduction in the protein concentration of extracts from neutral and high pH buffers was observed, heat treatment did not affect the solubility of proteins in the low pH buffers, particularly the citrate-5 buffer. A significant reduction in the protein content of extracts may be attributed to the denaturation and aggregation of some oyster proteins. Heat treatment unfolds the protein, exposing the hydrophobic residues from its structure and subsequently prompting the formation of insoluble aggregates [29]. Wet heat treatments can affect the solubility of proteins greatly as shown by Lasekan and Nayak [10] for shrimp allergens. While the effects of temperature on the solubility of proteins have been thoroughly studied, the ability of proteins to resist heat treatment at low pH solution is not well understood.

The composition of the extracts for different proteins was determined using high-resolution mass spectrometry enabling in-depth comparison of each extract. Mass spectrometry analysis showed the numbers of proteins identified were different in each extract. As expected from the protein quantification, low pH extracts contained fewer proteins as compared to the neutral or high pH extracts. Interestingly, although addition of a high salt concentration or high pH increased the total protein concentration, the numbers of proteins identified in their extracts were less, as compared to the normal TBS or PBS. These findings suggest that an increase in protein content in high salt or high pH buffers was mostly due to the increase in the abundance of specific proteins. Further analysis of each extract demonstrated that not only protein composition varied, but the composition of potential allergens was also different in each extract. In total, 38 potential allergens could be identified from the extracts. Interestingly, the common buffers, TBS and PBS, extracted more potential allergens than other buffers. However, the abundance of these potential allergens in those buffers is often low, affecting the IgE-reactivity as a result.

The effect of buffers on the soluble proteins was evident after resolving the proteins in the polyacrylamide gels. Three distinct protein profiles were observed; while the neutral and high pH buffers showed a similar pattern of protein profiles, the low pH buffers exhibited distinct protein profiles. Some proteins were extracted better by high salt buffers or high pH buffers compared to other buffers as shown by the increase of protein staining intensity in the SDS gels. The change in protein content and abundance in turn affects the number of IgE reactive bands observed, as additional IgE-reactive bands were revealed in the TBSN, PBSN and Carbonate-10 extracts. This corresponds to the SDS-PAGE bands with higher intensity as compared to the PBS or TBS extracts.

The serum IgE analysis by immunoblotting demonstrated the superiority of the high salt or high pH carbonate buffers in solubilising less abundant but highly immunoreactive proteins as compared to the general buffers. One of the very prominent IgE-reactive bands is paramyosin, observed at 100 kDa. Paramyosin is a major structural component of the invertebrate muscle thick filament and was identified as an additional major allergen in abalone (*Haliotis discus discus)* [7,30] and recently in sea snail (*Rapana venosa*) [31]. The discovery of allergenic paramyosin in mollusc species was not surprising since this protein has been confirmed as a major allergen in other invertebrates such as house dust-mite [32] and anisakis [33]. Furthermore, this protein also forms a significant component of the bivalve myofibril with 38–48% in the white adductor muscle and 15–30% in the red adductor muscle [34]. However, paramyosin has a poor solubility in low ionic strength buffers, thus a high concentration of salt is required to adequately extract this protein. Moreover, the structural stability of this protein is susceptible to heat treatment, further affecting its IgE-binding capacity. A recent study by Yu et al. confirmed our finding that paramyosin is not heat stable, and we verified this using different buffers [31]. Nonetheless, it was observed in the Carbonate-10 extract that paramyosin content was relatively high and its IgE-binding capacity was still maintained following heat treatment. The protective mechanism of a high pH buffer on paramyosin stability is unknown. It is postulated that at high pH, the net charge of the protein increases enhancing the electrostatic repulsion between protein molecules to reduce their aggregation in the aqueous solution [35].

Four of eight IgE-reactive bands in this study were heat stable proteins including bands at about 39, 50, 100 and >200 kDa. The 39 kDa IgE-reactive protein was identified as tropomyosin and has been previously identified as a major allergen in various mollusc species including squid [36], oyster [37] and abalone [38]. Tropomyosin is a heat-stable and water-soluble protein, and due to its abundance in muscle tissue, the extraction process for this protein is relatively easy. Tropomyosin was also observed at the 50 kDa IgE-reactive spot with a high Mascot score and sequence coverage consistent with heat-induced degradation and aggregation. This higher molecular weight tropomyosin was also observed in other species including Sydney rock oyster [19] and Black tiger prawn [39]. In summary, each band contained between 2–4 known allergenic proteins, which could all contribute to the IgE reactivity. While many proteins are identified at their respective molecular weight bands (e.g., tropomyosin, arginine kinase, paramyosin), some allergens seem to be present as protein fragments/aggregates, which might still have IgE binding capacity (e.g., myosin heavy chain, filamin).

## 5. Conclusions

In conclusion, buffer compositions affect considerably the protein recovery during extraction from oyster tissue, resulting in variation of IgE-reactive proteins. Many allergens are often overlooked during allergen discovery analysis due to low abundance, as the common buffers used for protein recovery are unable to sufficiently extract certain allergenic proteins. This study is the first to investigate in detail the extractability of animal allergens and demonstrated that increasing ionic strength or pH improves the extractability of the buffers, allowing much greater discovery of IgE binding proteins.

## Figures and Tables

**Table 1:** Buffers and their composition used to extract proteins from Pacific oyster.

Buffer	pH	Composition
Citrate-3	3.0	Citric acid 0.082 M Trisodium citrate 0.018 M
Citrate-5	5.0	Citric acid 0.065 M Trisodium citrate 0.035 M
TBS	7.4	Tris 25 mM Potassium Chloride 3.0 mM Sodium Chloride 140 mM
TBSN	7.4	Tris 25 mM Potassium Chloride 3.0 mM Sodium Chloride 1 M
PBS	7.4	Phosphate 10 mM Potassium Chloride 2.7 mM Sodium Chloride 137 mM
PBSN	7.4	Phosphate 10 mM Potassium Chloride 2.7 mM Sodium Chloride 1 M
Carbonate-9	9.2	Sodium Carbonate 0.01 M Sodium Bicarbonate 0.09 M
Carbonate-10	10.3	Sodium Carbonate 0.07 M Sodium Bicarbonate 0.03 M

**Table 2:** Demographics of patients recruited for this study.

	Sex	Age (yrs)	Total IgE (kU/L)	Specific IgE (ImmunoCAP kU/L)			Skin Prick Test		
				Oyster (f290)	Shrimp (f24)	HDM (d1)	Shrimp	Oyster	HDM
1	M	50	976	2.04	9.03	13.60	NT	NT	12 mm
2	F	28	461	0.11	0.36	54.8	NT	NT	6 mm
3	M	43	194	NT	1.41	0.35	10 mm	3 mm	10 mm
4	F	38	28	3.75	9.82	2.66	NT	NT	0 mm
5	M	38	183	1.04	6.84	31.70	NT	NT	NT

Note: NT = Not Tested.

**Table 3:** The yield of recovered proteins measured by BCA–protein quantification method. Protein concentration was statistically analysed by one-way ANOVA (Tukey).

Buffer	Protein Concentration (mg/mL)	
	Raw	Heated
Citrate-3	2.26 ± 0.29 ^a^	2.35 ± 0.39 ^a,b^
Citrate-5	3.04 ± 0.14 ^a^	2.42 ± 0.09 ^a^
TBS	6.99 ± 0.28 ^b^	1.61 ± 0.05 ^c^
TBSN	9.08 ± 0.29 ^c^	1.86 ± 0.05 ^c,d^
PBS	7.69 ± 0.23 ^b,d^	1.74 ± 0.04 ^c,e^
PBSN	9.70 ± 0.97 ^b,e^	2.07 ± 0.08 ^b,d,e,f^
Carbonate-9	8.04 ± 0.16 ^d^	1.88 ± 0.14 ^c,f^
Carbonate-10	10.43 ± 0.52 ^e^	4.29 ± 0.13 ^g^

^a,b,c,d,e,f,g^ Values with the same superscript letter in the same column are not significantly different (*p* > 0.05). Different superscript letters within the same column indicate significant difference (*p* < 0.05).

**Table 4:** Proteins identified using LC-MS in the SDS-PAGE bands corresponding to the IgE-reactive bands. The top three proteins from Mascot search engine result in each band are presented and ordered based on their abundance in the spot.

Band No	Protein	Accession ID	Exp MW	Theo MW	Mascot Score	Coverage (%)	Number of Significant Peptides	emPAI
**Raw**								
1	Tropomyosin	B7XC66_CRAGI	36	33	1566	62	19	22.48
	Myosin heavy chain	K1RSS3_CRAGI		230	1463	20	33	1.42
	Filamin	K1PW06_CRAGI		326	553	12	6	0.23
2	Arginine kinase	K1PLF9_CRAGI	39	40	1749	72	23	34.84
	Tropomyosin	B7XC66_CRAGI		33	1601	55	19	20.33
	Filamin	K1PW06_CRAGI		326	2533	32	65	1.17
3	Retinal dehydrogenase I	K1QVG5_CRAGI	50	53	1256	59	43	6.72
	Aldehyde dehydrogenase	K1QNT7_CRAGI		58	648	42	17	3.01
	Tubulin α-1C chain	K1QII6_CRAGI		51	744	47	14	2.74
4	Paramyosin	K1QTC1_CRAGI	100	98	6288	74	69	61.54
	Alpha-actinin	K1RH58_CRAGI		102	1480	56	38	4.44
	Filamin	K1PW06_CRAGI		326	1711	28	50	1.00
5	Filamin	K1PW06_CRAGI	150	326	7159	61	151	7.06
	Clathrin heavy chain	K1PNR3_CRAGI		193	1660	44	60	2.22
	Spectrin α chain	K1R401_CRAGI		287	1905	45	77	1.69
**Heated**								
6	Tropomyosin	B7XC66_CRAGI	36	33	1570	59	21	33.41
	Myosin heavy chain	K1RSS3_CRAGI		230	1988	27	44	1.56
	Filamin	K1PW06_CRAGI		326	635	16	23	0.32
7	Tropomyosin	B7XC66_CRAGI	39	33	3535	57	21	60.09
	Troponin T	K1QPC9_CRAGI		21	522	74	10	7.17
	Non-neuronal cytoplasmic intermediate filament protein	K1PBC0_CRAGI		70	410	35	15	1.19
8	Tropomyosin	B7XC66_CRAGI	50	33.1	822	52	13	5.77
	Non-neuronal cytoplasmic intermediate filament protein	K1PBC0_CRAGI		69.6	1599	49	29	5.32
	Adipophilin	K1PJC1_CRAGI		54.4	791	41	16	2.44
9	Paramyosin	K1QTC1_CRAGI	100	98	7125	63	46	20.17
	Filamin	K1PW06_CRAGI		326	5441	47	107	2.60
	Myosin heavy chain	K1RSS3_CRAGI		230	4644	45	115	1.71
10	Myosin heavy chain	K1R1B3_CRAGI	120	80	3998	58	36	11.60
	Paramyosin	K1QTC1_CRAGI		98.1	1523	45	36	2.15
	Filamin	K1PW06_CRAGI		326.2	1796	23	53	0.73

## Data Availability

Not applicable.
